# A Highly Accurate Positioning Solution for C-V2X Systems

**DOI:** 10.3390/s21041175

**Published:** 2021-02-07

**Authors:** Qi Liu, Peng Liang, Junjie Xia, Ti Wang, Meng Song, Xingrong Xu, Jiachi Zhang, Yuanyuan Fan, Liu Liu

**Affiliations:** 1 China Unicom Smart City Research Institute, Beijing 100033, China; liuqi49@chinaunicom.cn (Q.L.); liangp@chinaunicom.cn (P.L.); xiajj2@chinaunicom.cn (J.X.); wangti2@chinaunicom.cn (T.W.); songmeng@chinaunicom.cn (M.S.); xuxr10@chinaunicom.cn (X.X.); 2 School of Electronic and Information Engineering, Beijing Jiaotong University, Beijing 100044, China; jiachi_zhang@bjtu.edu.cn (J.Z.); 19120048@bjtu.edu.cn (Y.F.)

**Keywords:** cellular vehicle-to-everything, vehicular positioning, feasibility and challenges, network architecture

## Abstract

Cellular vehicle-to-everything (C-V2X) is essential in enabling safe, reliable, and efficient transportation services. It serves as serve as the foundation for vehicles to communicate with each other and everything around them. One fundamental element in C-V2X is positioning, namely extracting the vehicle’s absolute and relative positions concerning other objects such as buildings, pedestrians, traffic signs, and other vehicles. However, its feasibility in enabling vehicular positioning has not been fully explored yet. In this paper, key performance indicators (KPIs) for C-V2X positioning have been described firstly. Then positioning challenges and conventional positioning methods for C-V2X are reviewed. Afterward, two user equipment (UE)-based and UE-assisted C-V2X positioning architectures are proposed, and key technologies are also described. Lastly, testing and typical application cases are provided.

## 1. Introduction

The transportation is considered as one of the most significant economic sectors and sources of income in the world. In recent years, automotive, communications companies, academics, and the government in the world, are developing a novel vehicle communication technology. It is termed as the vehicle-to-everything (V2X), which can provide a real-time and highly reliable information data pipe to enable safe, efficient, and economical transportation services and provide the possibility to connect and autonomous driving [1,2,3]. The original V2X communication technology, i.e., the DSRC is based on the IEEE Standard (Std.) 802.11p. Ever since the initial release of DSRC in 1999, various V2X technologies have been developed to support ubiquitous, wide-scale, and high-performance communication methods for vehicle users [4]. Cellular V2X (C-V2X) employs 3GPP standardized 4G LTE or 5G mobile cellular communication system to establish a wireless link from a vehicle to other vehicles, pedestrians, or to other fixed objects such as buildings, pedestrians, traffic signs in its surroundings. Compared with another vehicular communication system, DSRC (802.11p), C-V2X can provide different applications with low-latency, high-reliability, and high-throughput. Besides, the established base-stations of 4G LTE or 5G also can be shared. In most countries such as America, European countries, Japan, and China, 5.9 GHz frequency band is officially assigned for intelligent transportation system frequency [5,6,7].

**Figure 1:**
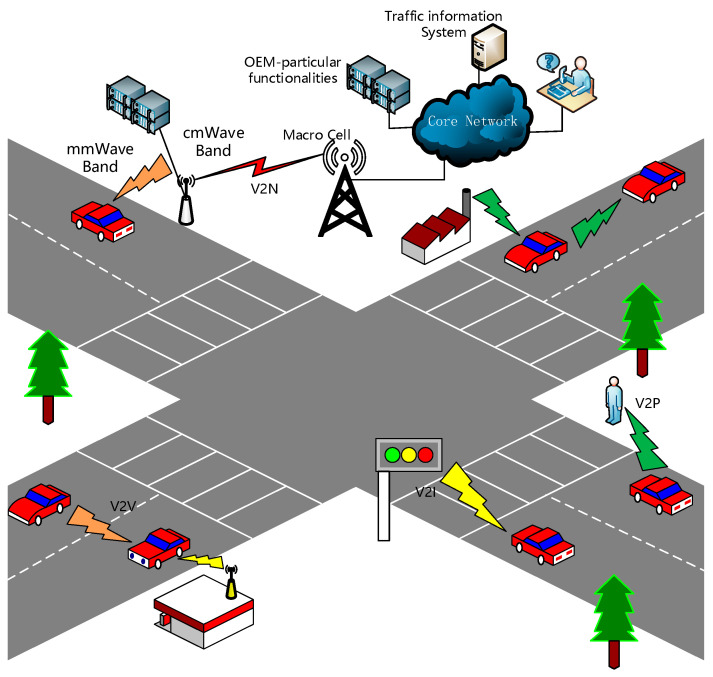
The scenario of Vehicle-to-Everything.

As shown in Figure 1, according to the participants of the communication, the vehicle communication Network can be categorized as:

- Vehicle-to-Vehicle (V2V): communicating directly with different moving vehicles;
- Vehicle-to-Infrastructure (V2I): communicating with infrastructures along the roadside;
- Vehicle-to-Pedestrian (V2P): communicating with pedestrians in the vehicle’s surrounding;
- Vehicle-to-Network (V2N): communicating with IT networks and/or data centers.

There are various use cases involved in the V2X services, e.g., emergency brake warning, pre-crash sensing warning, road hazard warning, congestion alert, speed guiding, autonomous driving, remote driving, etc. [8,9,10]. Thus, one fundamental element in C-V2X is positioning, namely recognizing the car’s absolute and relative positions concerning other surrounding objects such as vehicles, pedestrians, traffic signs, and other buildings. In particular, autonomous driving, the most exciting application in C-V2X, places much more stringent requirements on positioning accuracy than other services because an error can lead to fatal accidents [11,12].

The main contribution of this paper is to investigate the highly accurate positioning solution for C-V2X systems. Based on key KPIs of the positioning system and positioning challenges for V2X, we propose two system architectures for C-V2X high accuracy positioning from the telecom operator point of view as:

(1) UE-based Positioning Architecture:

UE-based positioning is that the positioning calculation is completed at the terminal side by using the assisted information providing by the network such as RTK, map data, etc. For the network, it’s mainly used on RTK data transmitting and 5G-based positioning. And for the terminal, it’s mainly used on RTK positioning and fusion algorithm.

(2) UE-assisted Positioning Architecture:

UE-assisted positioning is that the positioning calculation is completed on the network side by collecting all the information from both the road side and terminal side. For the network, it is mainly used on RTK positioning, 5G-based positioning, fusion algorithm and positioning results transmitting. For the terminal, it is mainly used on measurement report and positioning results receiving. And the positioning results can be transmitted to the terminal through eNB/gNB/RSU.

The rest of the paper is organized as follows. Section 2 provides KPIs for V2X positioning system, positioning challenges and conventional positioning methods for C-V2X. In Section 3, the system architecture for C-V2X high accuracy positioning is proposed. In Section 4, key technologies for high accuracy positioning of vehicles are described. Testing and typical application cases are provided in Section 5, and conclusions are drawn in Section 6.

## 2. Positioning Challenges and Conventional Positioning Methods for C-V2X

### 2.1. Important KPIs of Positioning for C-V2X

With the evolution of V2X services from assisted driving to autonomous driving, the use case requirements are also changing the availability of network coverage, level of uncertainty, availability of features for simultaneous localization and mapping (SLAM)-based positioning techniques, reliability, latency, speed, data rate, communication range, as well as positioning accuracy, which varies from meter level to sub-meter level. Some important KPIs are described as follows [13]:

- Position Accuracy: describes the closeness of the measured position of the UE to its true position value. The accuracy can describe the accuracy either of an absolute position or a relative position. It can be further derived into a horizontal position accuracy—referring to the position error in a 2D reference or horizontal plane, and into a vertical position accuracy—referring to the position error on the vertical axis or altitude.
- Availability: percentage of time when a positioning system can provide the required position-related data within the performance targets or requirements.
- Update rate: the rate at which the position-related data is generated by the positioning system. It is the inverse of the time elapsed between two successive position-related data.
- Reliability: the measure of the ability of a positioning system to provide the position-related data under stated conditions for a specified period.
- Latency: time elapsed between the event that triggers the determination of the position-related data and the availability of the position-related data at the positioning system interface. At the initialization of the positioning system, the latency is also defined as the Time to First Fix. Though it is capable of achieving centimeter-level accuracy and is widely used for mapping, its application to real-time positioning is problematic due to the scanning and processing latency.
- Power consumption: electrical power (usually in milliwatt) used by the positioning system to produce the position-related data.

The accuracy requirements are different according to different business scenarios. Table 1 shows some positioning accuracy requirements in different use cases for business scenarios involving information service, vehicle safety, and traffic efficiency [14].

### 2.2. Positioning Challenges for C-V2X

Currently, positioning requirements in C-V2X scenarios mainly meet the following three challenges: positioning requirements in different application scenarios, drawing and updating high-definition (HD) map, and cost of high accuracy positioning:

- To meet positioning requirements in different application scenarios. Currently, outdoor positioning technology is mainly based on real-time kinematic (RTK), which can achieve centimeter-level positioning in an open and unobstructed scenario. However, considering intensive buildings in the urban environment, as well as the occlusion scenes such as tunnels/viaducts/underground parking, it needs to be combined with the inertial unit to maintain the accuracy in a continuous time by using a fusion algorithm. Therefore, how to ensure long-term stable and high accuracy positioning of the vehicle in all scenarios is a great challenge in C-V2X application scenarios. It is necessary to ensure the positioning accuracy of the vehicle anytime and anywhere through multi-source data fusion, as combining the cellular network positioning, inertial navigation, radar, camera, etc. e.g., path planning and lane-level monitoring in C-V2X services require corresponding high accuracy map matched to ensure positioning accuracy.
- Costly high accuracy positioning. To ensure the performance requirements of high accuracy positioning of vehicles, it is necessary to integrate cellular networks, satellites, inertial navigation, cameras, and radar data. However, drawing HD maps is a high-cost and complex work, and it requires regular updates to ensure accurate and reliable positioning performance and service requirements. The high cost and difficulty of rapid popularization limit the business services of high accuracy positioning.

### 2.3. Conventional Positioning Methods for C-V2X

The positioning solutions are significantly different according to the positioning requirements and environments.

#### 2.3.1. GNSS (Global Navigation Satellite System) 

**Figure 2:**
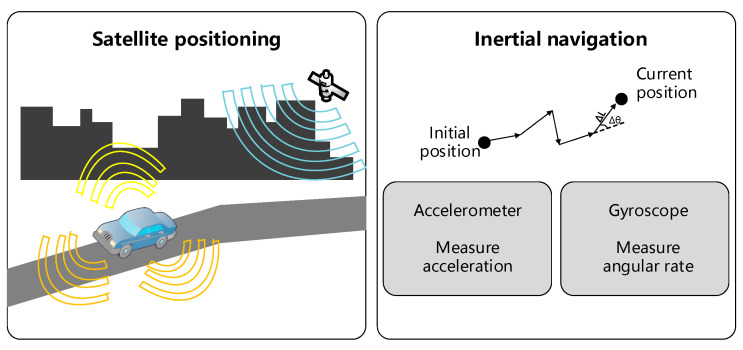
The work principle of the INS in vehicular scenarios.

GNSS uses satellites to extract real-time, exact, fast, and continuous three-dimension position location and velocity information as shown in Figure 2 [17,18]. GNSS or its differential complement RTK, is the most basic positioning method. Considering that GNSS is not available in indoor scenarios such as a tunnel or dense urban, its application scenario is limited to the outdoor environment. However, GNSS can deliver a position solution with accuracy on the order of several meters, which does not satisfy the stringent requirement for autonomous driving. Besides, multipath propagation and obscuration factors affect GNSS signals, which degrade the reliability and availability. For more accurate positioning of GNSS, two solutions are available:

(1) GNSS location service based on RTK differential system [19]

High accuracy GNSS enhancement technology implements satellite observation through the ground differential reference station to form differential correction data and then transmits the data to the flow measurement station through the data communication link, and then the flow measurement station locates according to the correction data received.

(2) GNSS location-based SSR services [20]

State-Space Representation (SSR) is a modern, high accuracy (centimeter-level) GNSS positioning technique which has been described in [21,22,23]. SSR differs from the observation space representation by enabling the individual ‘state’ of each error component to be modeled and sent at the optimum update rate, leading to bandwidth reductions and other advantages described below. The primary SSR correction messages supported in LPP are the satellite orbit, satellite clock, satellite code and phase biases, and the tropospheric and ionospheric corrections.

#### 2.3.2. Inertial Navigation System (INS) 

The inertial navigation system (INS) is a widely used navigation device without any dependence on external reference information [17,24]. It continuously calculates and estimates the position, the orientation, as well as the velocity (including direction and velocity) by utilizing a combination of computer, motion sensors (accelerometers), and rotation sensors (gyroscopes). Two core modules of the inertial sensors or inertial measurement units (IMU), i.e., accelerometers and gyroscopes, account for the measurements of linear accelerations and vehicle angular velocities, respectively. These data are cornerstones for further vehicle trajectory calculation as it moves and the vehicle position is being calculated relative to its corresponding initial position. INS is a self-contained system and unlike GNSS, its performance will not degrade due to the environment. However, INS performance degrades with time due to the accumulation of measurement errors and to limit the errors, regular updates from another source of positioning is necessary. With a capacity of self-contained navigation, INS can be integrated with other positioning technology (such as the prevailing GPS) to boost the reliability performance of a vehicle-positioning unit. INS presents good robustness to estimate the vehicle’s position even if the number of GPS and other positioning observations is less than four.

#### 2.3.3. Cellular Positioning 

In the cellular networks (such as GSM/UMTS/CDMA/LTE/NR and other wireless network systems), a dedicated positioning signal was put forward, which was named PRS [17,25]. The Base Station (BS) sends PRS in the downlink, and the UE calculates the arrival time differences of the PRS among different cells and then gets the positioning results through the positioning algorithms. The major drawback of such technology lies in the geographical coverage. Whereas it presents a merit of high accuracy in the location estimation within buildings or areas with good network coverage. This solution uses cell-ID, AoA (Angle of Arrival), TDoA (Time Difference of Arrival), ToA (Time of Arrival), and RSS (Received Signal Strength) to extract the wanted position. However, the cellular positioning technology can be positioned within the range of several tens of meters to several hundreds of meters. A 5G forum white paper recommends a latency accuracy on the order of 1ms for 5G positioning networks [26].

#### 2.3.4. Sensors and HD Map Matching Positioning 

**Figure 3:**
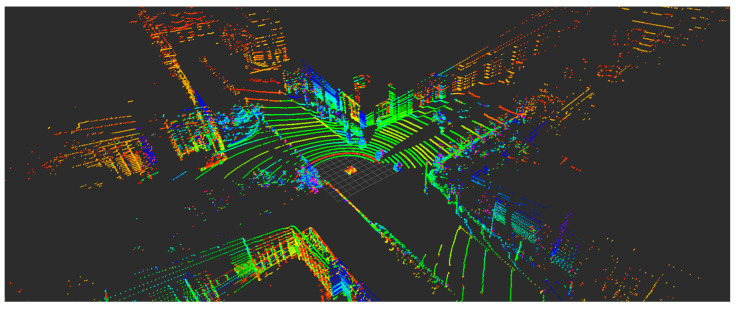
A cloud image of a vehicle approaching, an intersection illustrates the complexity of data collected by a LiDAR.

Positioning based on sensors and HD maps is another common positioning method for vehicles as shown in Figure 3 [27,28]. Two main techniques, SLAM and HD maps, are considered here. The SLAM-based techniques can reorganize as well as position in real-time by monitoring the surroundings continuously, which enables them to quickly adapt to new environments. Notably, these techniques demand a large number of computation-intensive algorithms. Besides, the sensors along with the surroundings may bring uncertainties to these systems. To handle these drawbacks, the HD map technique is proposed to provide a detailed description of the autonomous vehicle (AV) surroundings. It can identify the known objects quickly, assisting the perception task for AV systems dramatically. HD maps present a good positioning performance on the condition of the unchanged physical environment, but a degradation for autonomous driving will occur if the environment varies significantly. As such, it is essential for HD maps to update timely to offer a high location accuracy for the real-time perception of AVs. However, the quality of map data will decide the location accuracy as well as a series of usability features directly. The high costs and vulnerability to the environment also constrain its application prospect. Besides, the reliable transmission of real-time HD maps (large size) is challenging in time-varying channels.

Generally, one single technology such as GNSS or sensors is hard to satisfy the actual requirements to guarantee the positioning performance for C-V2X. All these techniques require a precise depiction of the physical environment space information. This information can be offered by a proper sensor selection, which refers to the number, types, capabilities, and spatial arrangement of sensors. However, different sensor technologies derive from different sources and yield various error characteristics. Thus, it is essential to investigate a rigorous sensor model to enhance the accuracy, reliability, and robustness of the positioning system. For example, the cellular network, apart from cellular positioning, is essential to enhance the positioning performance, such as the transmission of RTK data and the sensor data, download of HD map and even the fusion of the positioning data, etc. In contrast, C-V2X use different sensors, require real-time operations and are primarily interested in identifying and tracking moving objects. Corporative and combining positioning may be the optimal positioning solution to ensure high positioning accuracy.

## 3. System Architecture for C-V2X High Accuracy Positioning

Combining the scene analysis and performance requirements of C-V2X, high accuracy positioning, as a key part of the whole C-V2X system, its architecture can be divided into UE-based positioning architecture and UE-assisted positioning architecture.

### 3.1. UE-Based Positioning Architecture

**Figure 4:**
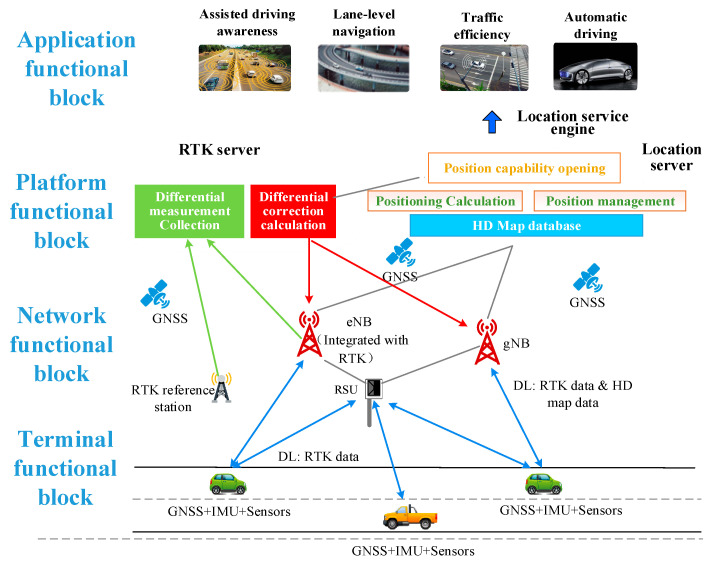
UE-based Positioning Architecture of V2X system.

The UE-based positioning architecture includes terminals, network, platform, and application functional blocks, as shown in Figure 4. The terminal functional block implements multi-source data fusion (satellite, sensor, and cellular network data) algorithms to ensure the positioning requirements of different services and scenarios. The platform functional block provides positioning platform functions for integrated vehicles, including RTK differential decomposition computing capability, map database, and dynamic HD map, positioning engine, and open positioning capability. Network functional block includes 5G BS, RTK BS, and roadside unit (RSU) to provide reliable data transmission for positioning terminal. Application functional block based on high accuracy positioning system can provide services such as lane-level navigation, line planning and autonomous driving. The signaling processing is plotted in Figure 4.

**Terminal functional block**

- Network functional block mainly implements signal measurement and information transmission, including the deployment of 5G BSs, RTK BSs, and RSU roadside units. As a new generation of communication technology, 5G ensures a high data transmission rate to meet the requirement of real-time transmission on high-precision maps. 5G BS can also complete the signal measurement with the terminal and report platform. Platform compute the positioning values based on 5G signal to assist in high accuracy positioning of vehicles. Based on 5G MEC (Mobile Edge Computing), the real-time update can be realized on HD maps. Real-time performance and accuracy will be greatly improved.
- Ground-based augmentation station is mainly used for RTK measurement and can be co-constructed with operator BS, which will greatly reduce the cost of network deployment, operation and maintenance. Meanwhile, the measurement data transmission of RTK BS can be realized through the 5G network, it will benefit from deploying the reference station quickly and flexibly.
- RSU can realize RTK information broadcast, which avoids the reporting of the terminal’s initial position in the traditional RTK positioning. Meanwhile, RSU provides local road lane level maps and real-time dynamic traffic information broadcasts.

**Platform functional block**

The platform functional block can be modularized, including:

- HD map. Static HD map information, such as lane lines, lane center lines, lane property changes, etc. In addition, it also includes road parameters such as curvature, slope, heading, and cross slope, which enables the vehicle to accurately turn, brake, climb, etc. It also includes road signs such as traffic and road signs, and special certain points such as areas where GPS disappears and road construction status.
- Dynamic traffic information, such as road traffic conditions, construction conditions, traffic accidents, traffic control, weather conditions, etc.
- Difference decomposition calculation. The platform continuously receives satellite data through RTK BS, and optimizes various major system error sources such as ionospheric error, tropospheric error, orbit error, and multipath effect, and establishes the error model of ionospheric delay and tropospheric delay for the whole network. The optimized spatial error will be sent to the vehicles.
- Data management, such as national administrative division data, vector map data, basic traffic data, massive dynamic emergency rescue vehicle position data, navigation data, real-time traffic data, points of interest (POI) data, etc. These data are operating data that have been integrated and compiled after the data production process.
- Data calculation, including path planning, map static data calculation, dynamic real-time data calculation, big data analysis, data management, and other functions.

**Application functional block**

- In application functional block, it provides users with services like map browsing, planning route display, data monitoring, and management functions, as well as location-based other car networking services, such as assisted driving and automated driving.

### 3.2. UE-Assisted Positioning Architecture

UE-assisted positioning is that the positioning calculation is completed on the network side by collecting all the information from both the road side and terminal side.

**Figure 5:**
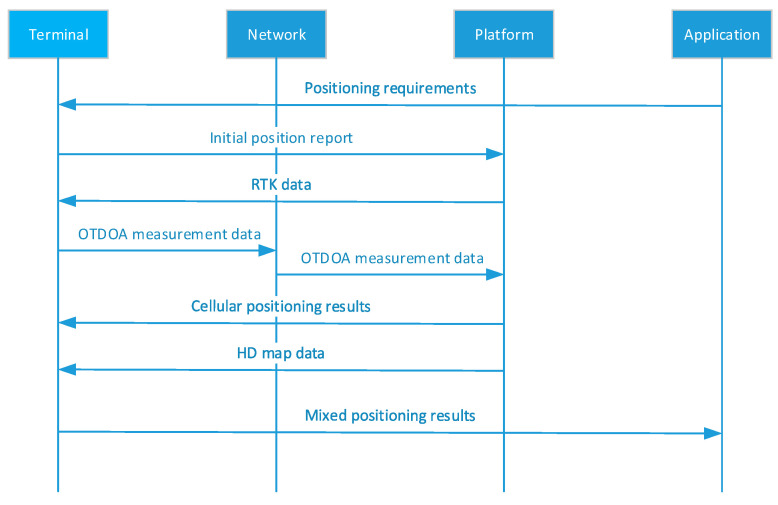
Signaling processing in the UE-based positioning architecture.

UE-assisted functions are presented in Figure 5. For the network, it is mainly used on RTK positioning, 5G-based positioning, fusion algorithm, and positioning results transmitting. For the terminal, it is mainly used on measurement reports and positioning results receiving. And the positioning results can be transmitted to the terminal through eNB/gNB/RSU.

**Figure 6:**
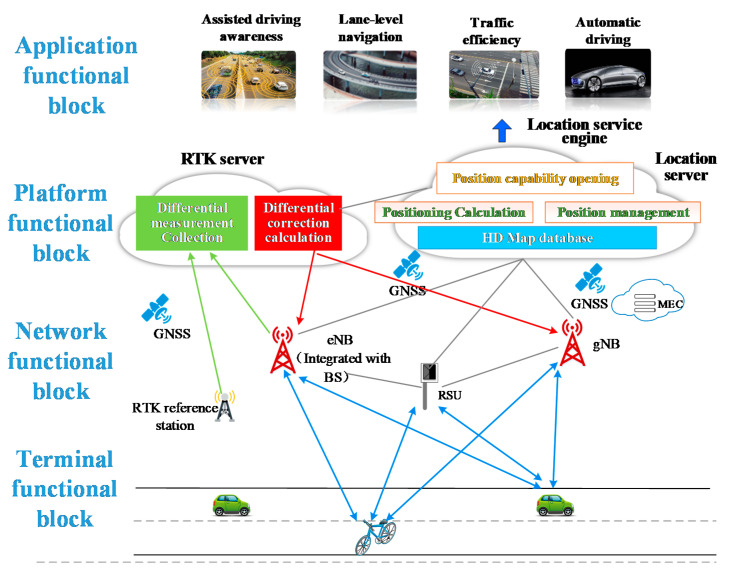
UE-assisted Positioning Architecture of V2X system.

Compared with Figure 6, the fusion algorithm is deployed at the network functional block, and the calculation can be realized quickly through MEC. Besides, typical UE-assisted positioning scenarios include VRU (Vulnerable Road Users) and traffic supervision. It decreases the terminal complexity and is suitable for high-precision positioning of pedestrians and non-motor vehicles in smart traffic. The signaling processing is plotted in Figure 5.

**Terminal functional block**

- To complete the position calculation on the platform, the terminal needs to transmit the measurement data to the platform including the GNSS data, the sensor data, and the inertial navigation data.

**Network functional block**

- Network functional block mainly implements signal measurement and information transmission, including the deployment of 5G BSs, RTK BSs, and RSU roadside units. As a new generation of communication technology, 5G ensures a high data transmission rate to meet the requirement of real-time transmission on high-precision maps. 5G BS can also complete the signal measurement with the terminal and report platform. Platform compute the positioning values based on 5G signal to assist in high accuracy positioning of vehicles.

**Platform functional block**

The platform functional block can be modularized, including:

- HD map. Static HD map information, such as lane lines, lane center lines, lane property changes, etc. In addition, it also includes road parameters such as curvature, slope, heading, and cross slope, which enables the vehicle to accurately turn, brake, climb, etc. It also includes road signs such as traffic and road signs, and special certain points like areas where GPS disappears and road construction status.
- Dynamic traffic information, such as road traffic conditions, construction conditions, traffic accidents, traffic control, weather conditions, etc.
- Difference decomposition calculation. The platform continuously receives satellite data through RTK BS, and optimizes various major system error sources such as ionospheric error, tropospheric error, orbit error, and multipath effect, and establishes the error model of ionospheric delay and tropospheric delay for the whole network. The optimized spatial error will be sent to the vehicles.
- Data management, such as national administrative division data, vector map data, basic traffic data, massive dynamic emergency rescue vehicle position data, navigation data, real-time traffic data, POI data, etc. These data are operating data that have been integrated and compiled after the data production process.
- Data calculation, including path planning, map static data calculation, dynamic real-time data calculation, big data analysis, data management, and other functions. The platform also completely adopt a positioning scheme mixing multi-source data in the terminal, including differential data-based GNSS positioning data, inertial navigation data, sensor data, HD map data, and cellular network data, etc.

**Application functional block**

**Figure 7:**
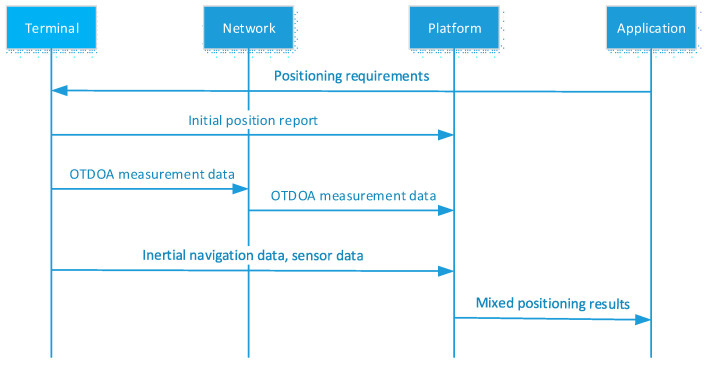
Signaling processing in the UE-assisted positioning architecture.

- The application functional block provides users with services as map browsing, planning route display, data monitoring, and management functions, as well as location-based other car networking services, such as assisted driving and autonomous driving. Detailed signaling processing information can be found in Figure 7.

## 4. Key Technology for High Accuracy Positioning of Vehicles

### 4.1. Sidelinking Positioning

The 3GPP has released the C-V2X standard to support V2X communications using the sidelink PC5 direct interface. The PC5 interface facilitates direct communication between two ends of a C-V2X communication link. The sidelink allows direct communication between two devices without going through a base-station. Since network infrastructure is not involved in the user-plane data transmission, packet transmission latency can be efficiently reduced [6].

The sidelink positioning is composed of three operations: the sidelink positioning configuration, the S-PRS transmission and measurement, the position calculation. The UE-based and UE-assisted positioning architecture in Uu link positioning are considered. First of all, the sidelink positioning is differentiated from Uu link positioning in that the sidelink positioning reference signal (S-PRS) transmission and measurement are done between UEs through sidelink interface. Second, the sidelink positioning scheduling and configuration can be controlled by the network (or location server) or UE itself that participated in the sidelink positioning process. Applying the positioning controllability to both UE-based and UE-assisted positioning architecture, totally four kinds of sidelink positioning architectures can be considered to be applied for both absolute and relative sidelink positioning.

#### 4.1.1. UE-Configured & UE-Based Sidelink Positioning Architecture

The target UE wants to know the position of itself, and the anchor UE as the UE or the RSU that participates in sidelink positioning and helps the target UE to acquire its position e.g., by sending/receiving S-PRS and doing relevant measurements.

In this architecture, all three sidelink positioning operations are done by the target or the anchor UEs that participate(s) in the sidelink positioning process. UEs form a sort of sidelink positioning group and send/receive S-PRS and calculate the target UE position based on the measurement done by UEs. Neither Uu link nor network-based location server is involved in the sidelink positioning process. It’s a fully distributed positioning based on the positioning group, which is comprised of a vehicle, a VRU, and a RSU. In one scenario using this architecture, the anchor UEs (e.g., RSU) can take the role of a location server, which are operated by e.g., a road operator.

#### 4.1.2. UE-Configured & UE-Assisted Sidelink Positioning Architecture

The main difference of this mode compared to the sidelink positioning mentioned above is the final positing calculation is done by the network or the location server, rather than the UEs of the sidelink positioning group, as the architecture name implies.

**Figure 8:**
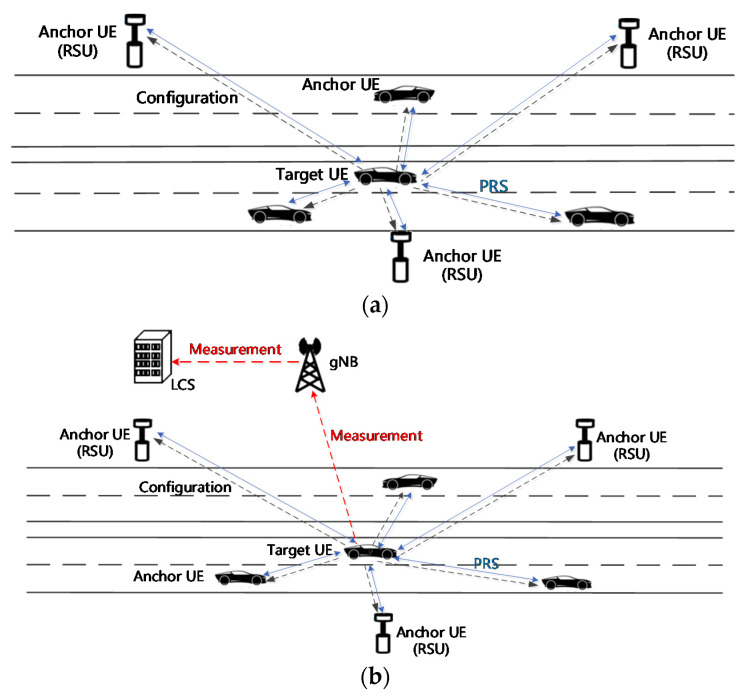
Two architectures of sidelink position: (**a**) UE-configured UE-based sidelink positioning architecture; (**b**) UE-configured UE-assisted sidelink positioning architecture.

The sidelink positioning configuration, as shown in Figure 8, the S-PRS transmission/reception through sidelink and the measurement are done by the UEs of the sidelink positioning group. However, the positioning measurement such as the RSTD or the angle of departure/arrival is reported to the network, and the final position of the target UE is calculated by the network or the location server. The pros of this architecture are offloading the position calculation to the network side, and a possible ultra-accuracy and faster position calculation based on the powerful processing power of the network. The cons are the increased latency to achieve the UE position.

### 4.2. Hybrid Data Fusion Method of Positioning of C-V2X

**Figure 9:**
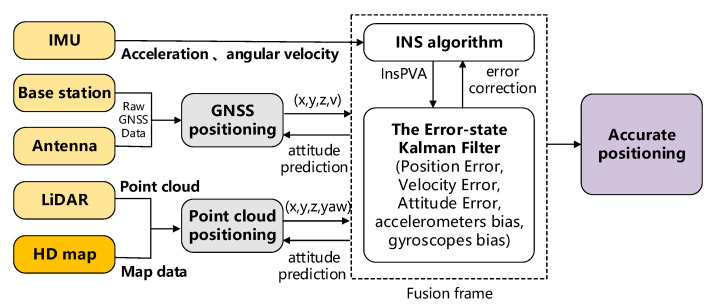
Kalman filter based hybrid data fusion method of positioning.

Demands for C-V2X accurate positioning based services have spurred many positioning techniques. However, due to the complex electromagnetic environments (non-line-of-sight propagation, multipath fading, and shadowing effects) and special application scenarios, the accuracy and robustness of the existing single system and technique do not perform well. To overcome this drawback, the hybrid data (GNSS, INS, cellular network data, and HD maps assisted sensor) fusion positioning of multiple positioning techniques have been proposed to improve the positioning performance in C-V2X, as shown in Figure 9. Hybrid data fusion combines data collected from different sources such that the final positioning result is in some sense better, more accurate, more complete, or more reliable than would be possible when these sources were used individually. In [29], a Kalman filter was utilized to fuse GPS, monocular vision, and HD map to enhance vehicle localization.

### 4.3. Synchronization for Positioning

Synchronization systems are an important part of most, although not all, (e.g., not part of multi-round trip time (RTT) mechanism) high-accuracy positioning systems, including both satellite navigation positioning and the ground high-accuracy positioning system. Every 3 ns decrease in the synchronization accuracy of a high-accuracy positioning system will lead to a range error of about 1 m. Therefore, synchronization performance has become a key index of the high-accuracy positioning system, and the high-accuracy synchronization technology of/between ground positioning network elements is the key to this field. V2X needs to meet the information exchange requirements of future intelligent driving, and the need for synchronization is obvious.

Since the positioning accuracy should be within 3~5 m to meet most positioning requirements of future intelligent transportation, and a margin should be considered for the measurement error, the synchronization accuracy of about 3ns~10ns is needed to realize the positioning accuracy of 3 m or even meters for the operator level ground positioning network.

### 4.4. Vehicular Positioning Using 5G Millimeter Wave

High accuracy positioning and the kinematic state of the vehicle are essential information for safety applications. When it comes to radio-based positioning techniques, huge improvements in massive MIMO, millimeter-wave (mmWave), introduced by 5G systems enhance the accuracy, latency, and reliability of C-V2X positioning [11]. The reasons are multiple. The large bandwidth available to mmWave frequencies enables a more accurate estimation of time of arrival (TOA) or time difference of arrival (TDOA) measurements. For mmWaves, more antennas can be packed in the same area, termed as beamforming, and this enables accurate estimation of the angle of departure (AOD) and angle of AOA. Hence, the combination of AOD or AOA with TOA makes position estimation more accurate compared with a single anchor mmWave systems. Studies on theoretical error bounds have shown that mmWave systems are capable of locating a UE with sub-meter positioning error and sub-degree orientation error [30].

## 5. Testing and Typical Application Cases

The following testing and typical application cases were conducted to verify the feasibility of our proposed methods. The RTK system based on the cellular network (Case 1) is to provide the industrial accuracy level of the RTK system based on the cellular network. The vehicle-pedestrian anti-collision case (Case 2) is an example of our proposed UE-based positioning architecture of V2X system. The parking management based on 5G high accuracy positioning (Case 3) is implemented on the cellular positioning system. The V2X application based on high-precision positioning (Case 4) is an example of corporative and combining positioning solution of GNSS, RTK, IMU, and high-precision map positioning methods.

### 5.1. Evaluation of RTK Systems Based on Cellular Networks (Case 1)

**Figure 10:**
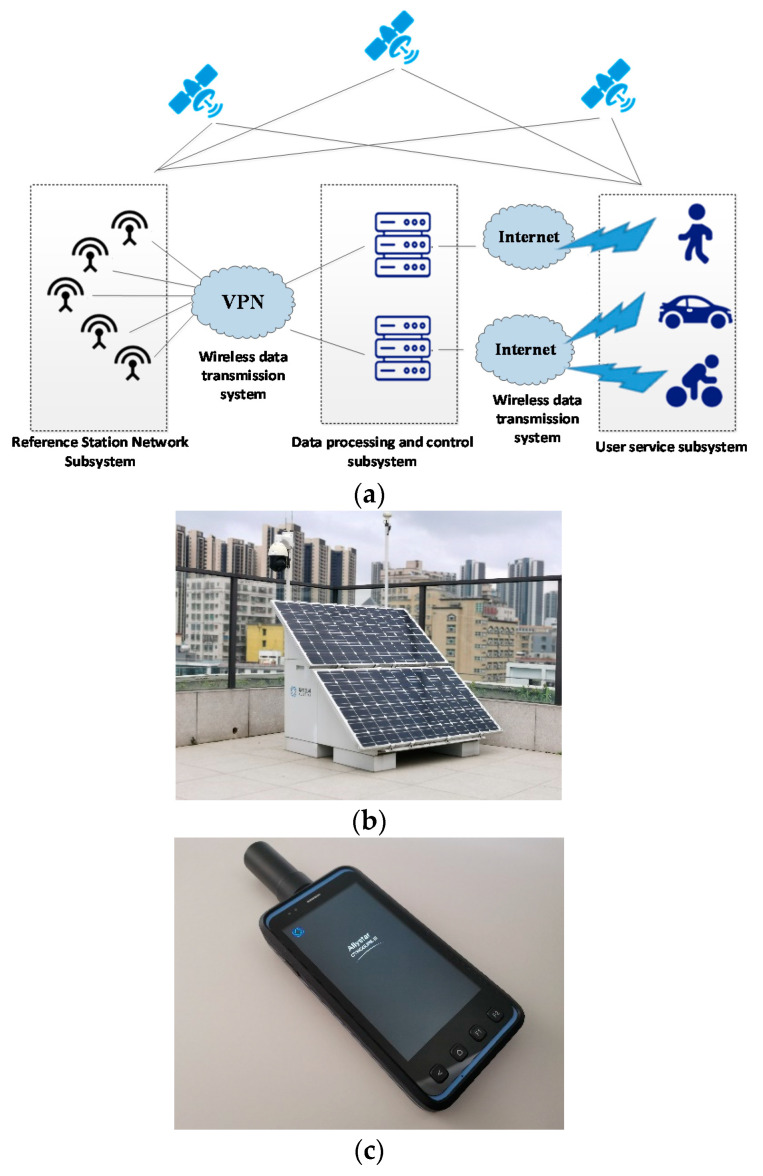
Evaluation of RTK systems based on cellular networks. (**a**) RTK network architecture diagram based on cellular network; (**b**) Photo of a reference station; (**c**) Photo of user device.

The RTK system based on the cellular network mainly completes the rapid deployment of reference stations, seen as Figure 10a. Through the wireless network backhaul, the rapid and flexible deployment of reference stations can be achieved without being restricted by the distribution of optical fibers. In this system, the data received by the reference station (Figure 10b) is transmitted to the server through the wireless communication system. Then the server implements the data processing and sends the values to the user terminals (Figure 10c). The functions of each part are shown in Table 2.

The RTK network elements based on cellular networks is composed of four subsystems, Reference station network subsystem, data processing and control subsystem, wireless data transmission system, and user service subsystem, as listed in Table 2. The step-by-step working processing of this system is as follows:

(1) Satellite positioning data acquisition: Multiple reference stations simultaneously collect GNSS satellite observations and send them to the control center. All reference stations should use geodetic-grade multi-frequency GNSS receivers.
(2) Data and control processing: The GNSS positioning data are then transmitted to data processing and control subsystem via the VPN data link. This subsystem will check the working conditions of the reference station, remotely control the reference station and users. Meanwhile, it will analyze, process, store and manage the data and form the data file in a certain format and send it to the user.
(3) User positioning: Users through operation service software and provide high-precision services.

**Figure 11:**
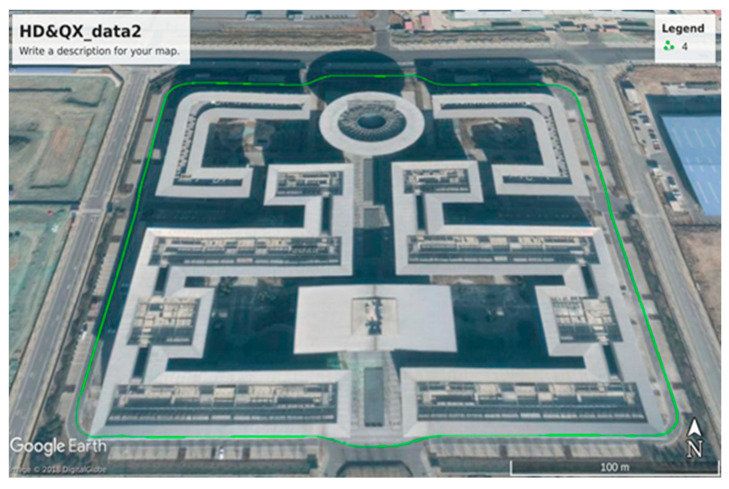
Dynamic test effect chart.

The test was carried out in Beijing (China). Two positioning boards of the same model and version were used for the experiments as shown in Figure 11. A pair of mushroom-head antennas were employed and connected to two devices through a power divider. One device used a commercial differential service system and one device used the rapidly deployed differential system. Two antennas were placed on the top of the same car, and the positioning error is about 10 cm.

**Figure 12:**
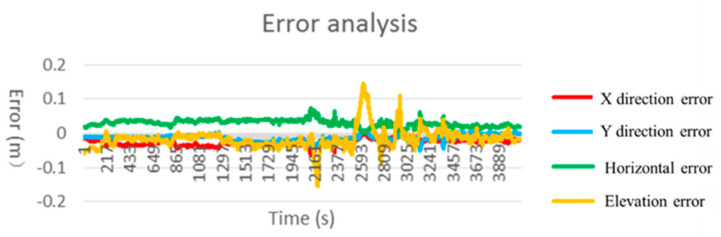
Error analysis chart.

Figure 12 plots the error result of the system. It can be found that the positioning accuracy is less than 0.2 m. In the results, the elevation error $e_{E}$ is less then horizontal value $e_{H}$. This is because $e_{H}=\sqrt{e_{x}^{2}+e_{y}^{2}}$, where the $e_{x}$ and $e_{y}$ are error values in *x* and *y* axis directions, respectively. The elevation error $e_{E}$, $e_{x}$ and $e_{y}$ are of the same order of magnitude. In addition, statistics on network performance show that the minimum delay is 199 ms, the maximum delay is 603 ms, and the average delay is 333 ms. The probability of less than 300 ms is about 66% and the probability of less than 500 ms is about 68%.

### 5.2. Vehicle-Pedestrian Anti-Collision (Case 2)

**Figure 13:**
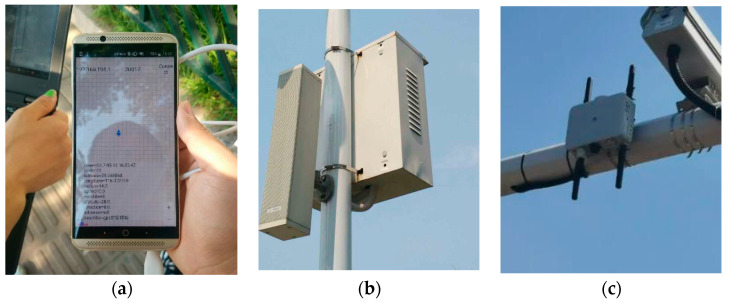

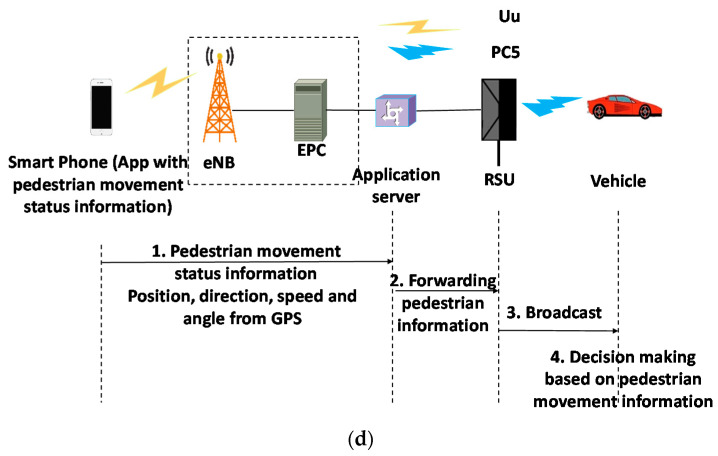
Vehicle-pedestrian anti-collision. (**a**). Photo of movement status information from the smart terminal; (**b**). Photo of eNB in the test; (**c**). Photo of RSU in the test; (**d**). Communication process of vehicle-pedestrian anti-collision.

This case is an application example of the UE-based positioning architecture of V2X system. The anti-collision is achieved using the exchanging of the accurate positioning information between the pedestrian and the car, but the conventional method (image recognition technology). In this vehicle-pedestrian anti-collision solution as shown in Figure 13d, the main processing is:

(1) The pedestrian movement status information, coming from GPS, is gathered with the smart terminal, as shown in Figure 13a.
(2) The positioning information is transmitted to the application server through LTE network, as shown in Figure 13b. The positioning information is then updated in database.
(3) The application server sends the data to the RSU (Figure 13c), and RSU broadcasts the movement status information to vehicles at intersections.
(4) After receiving the information of pedestrian movement, the vehicle will make a decision based on the collision avoidance algorithm, the buzzer and screen warning will be triggered if the pedestrian is in the area with collision risk.

This anti-collision solution was achieved based on China Unicom’s LTE network, high accuracy positioning, tracking technology, and LTE-V2X equipment and application server.

### 5.3. Parking Management Based on 5G High Accuracy Positioning (Case 3)

**Figure 14:**
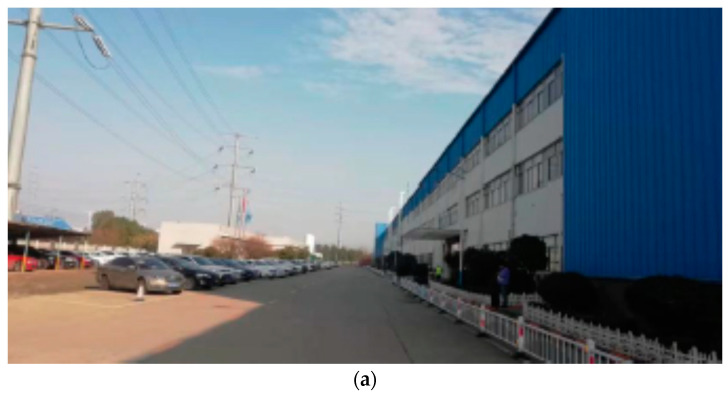

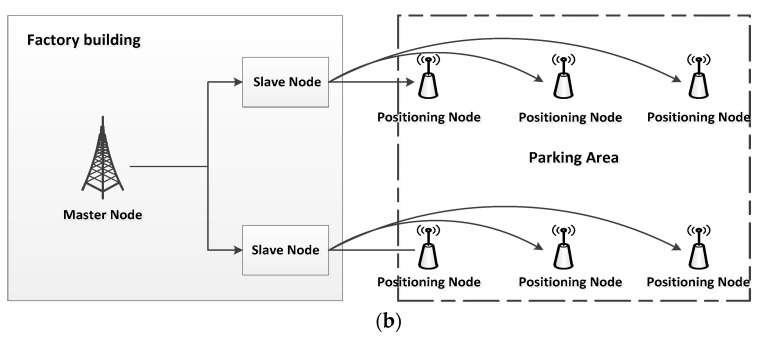
Parking management based on 5G high accuracy positioning. (**a**) Photo of the test site; (**b**) Deployment of 5G high accuracy positioning.

In 2019, a 5G-based high-accuracy positioning demonstration was conducted in the industrial areas as shown in Figure 14. By deploying 6 positioning base stations in the parking area of the industrial park, an overall coverage of the parking area (320 m × 50 m) by positioning service based on 3.5 GHz band signal is realized.

This solution achieved accurate positioning of 44 parking spaces. Through the statistics of dozens of positioning results for each parking space, the accuracy rate is higher than 95%, i.e., the probability of less than plus or minus 1.4 m error is more than 95% with the 2.8 m width of parking space, which can meet the demand of parking space management in the industrial park. In addition, the real-time tracking of the vehicle and the residence time recording of the vehicle in the parking space were also carried out.

### 5.4. V2X Application Based on High-Precision Positioning (Case 4)

In 2019, a test jointly demonstrated the V2X overall solution of high-precision map + high-precision positioning was conducted in the Wuxi car-connected city-level demonstration area.

**Figure 15:**
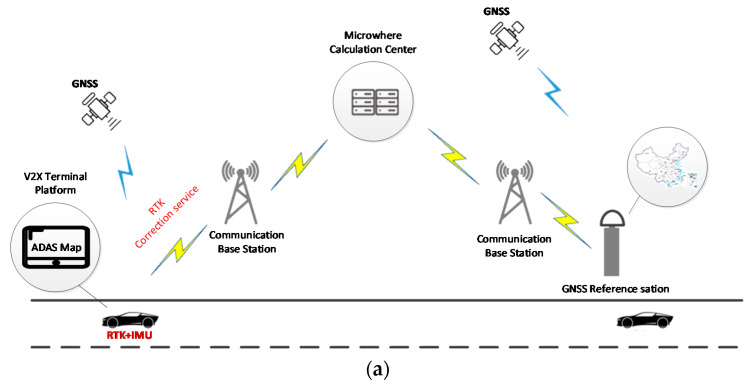

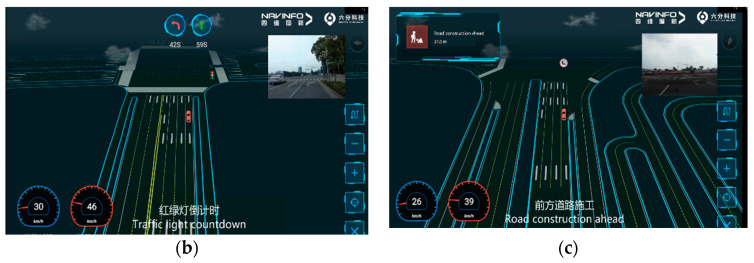
RTK + IMU integrated positioning solution.

In the joint test around the road about 10km, as shown in Figure 15a, the high-end positioning system at the vehicle used the GNSS RTK and IMU integrated positioning solution to output the required position, attitude, speed, and time information of the vehicle. RTK correction service used the GNSS reference station ground enhancement system deployed in the area to track GNSS satellites in the field of vision, and through centralized data processing and classification to obtain error correction parameters and integrity information, which was sent to the host through the communication network. Vehicle GNSS terminal equipment, thereby improving vehicle positioning accuracy and maintaining real-time lane-level positioning. RTK + IMU integrated positioning method provided integrated navigation for complex urban environments, and it also provided continuous and reliable high-precision positioning results in weak satellite signal coverage scenarios, such as overhead occlusion, short tunnels, and urban canyons.

RTK correction service guaranteed the improvement of vehicle position accuracy, thereby ensuring the accurate realization of V2X applications in positioning, and improving vehicle driving safety and road traffic efficiency. And GNSS RTK positioning could achieve more scene applications by matching with high-precision maps. In addition, by using this accurate positioning information, the special traffic conditions (traffic light, road construction or pedestrian) ahead also could be recognized, as shown in Figure 15b,c.

In addition, the KPI values of testing and typical application cases is listed in Table 3, which are informative for designing and optimizing V2X systems.

## 6. Conclusions

With the evolution of V2X services from assisted driving to autonomous driving, the use case requirements are also changing in reliability, latency, speed, data rate, communication range, as well as positioning accuracy, which is changing from meter level to sub-meter level. It is essential for autonomous driving to guarantee high accuracy positioning for both safety and reliability. In this paper, requirements, architecture, key technologies, and demonstrations in high accuracy positioning for V2X services are described. By combining the scene analysis and performance requirements, two system architectures for V2X high accuracy positioning are presented: UE-based and UE-assisted C-V2X positioning architectures. Besides, the key technologies for high accuracy positioning of vehicles are proposed to provide a reliable location service. Finally, we demonstrate the proposal through a series of tests, including the evaluation of RTK systems based on cellular networks, vehicle-pedestrian anti-collision, parking management based on 5G high accuracy positioning, and V2X application based on high-precision positioning. The results are beneficial and informative when designing a precise positioning system for C-V2X systems.

## Figures and Tables

**Table 1:** Positioning accuracy requirements in different use cases.

Business Type	Use Case	Communication Type	Positioning Accuracy (m)
Information Service	Road-user charging [15]	V2V, V2I	[1~3]
	Map download	V2N	≤[10]
	Ecall	V2N	≤[10]
	Parking guiding	V2V, V2P, V2I	≤[2]
Vehicle Safety	Pre-crash sensing warning [16]	V2V, V2I	≤[1.5]
	Emergency brake warning [16]	V2V	≤[1.5]
	Road hazard warning [16]	V2I	≤[5]
	Automated driving	V2V, V2N	≤[1]
	Remote driving	V2N	≤[1]
Traffic Efficiency	Congestion alert [16]	V2I, V2N, V2V	≤[5]
	Wrong way driving warning	V2I	≤[5]
	Speed guiding [16]	V2I	≤[5]
	Giving way for high priority vehicles	V2V, V2I	≤[5]

**Table 2:** RTK network elements based on cellular networks.

Subsystem	Function
Reference station network subsystem	A network of reference stations with ranges is usually less than 100 km. A network of reference stations usually requires a minimum of three reference stations to generate corrections for the network area.
Data processing and control subsystem	In this subsystem, the station observations are processed in a common network adjustment and observation errors and their corrections are computed
Wireless data transmission system	Reference station network: connects the reference station subsystem with the data processing and control subsystem, transmits the data including GNSS observation data and remote control data; User Network: Obtaining the differential data from the data processing and control subsystem through the cellular network
User service subsystem	Manage users through operation service software and provide GPS/Beidou high-precision services

**Table 3:** Key KPIs of Testing and Typical Application Cases.

KPI	Case 1	Case 2	Case 3	Case 4
Position Accuracy	<50 cm	<1 m	<1 m	<30 cm
Availability	>99.999% outdoor	>99.999% outdoor	>99.999%	>99.999% outdoor
Update rate	1 s	1 s	100 ms	1 s
Reliability	>99.999%	>99.999%	>99.999%	>99.999%
Latency	<200 ms	≤10 ms	≤10 ms	≤10 ms

## Data Availability

The data presented in this study are available on request from the corresponding author.
